# Placing human gene families into their evolutionary context

**DOI:** 10.1186/s40246-022-00429-5

**Published:** 2022-11-11

**Authors:** Alex Dornburg, Rittika Mallik, Zheng Wang, Moisés A. Bernal, Brian Thompson, Elspeth A. Bruford, Daniel W. Nebert, Vasilis Vasiliou, Laurel R. Yohe, Jeffrey A. Yoder, Jeffrey P. Townsend

**Affiliations:** 1 Department of Bioinformatics and Genomics, UNC-Charlotte, Charlotte, NC USA; 2 Department of Biostatistics, Yale School of Public Health, New Haven, CT USA; 3 Department of Biological Sciences, College of Science and Mathematics, Auburn University, Auburn, AL USA; 4 Department of Environmental Health Sciences, Yale School of Public Health, New Haven, CT USA; 5 Department of Haematology, University of Cambridge School of Clinical Medicine, Cambridge, UK; 6 European Molecular Biology Laboratory, European Bioinformatics Institute, Hinxton, UK; 7 Department of Environmental Health, Center for Environmental Genetics, University of Cincinnati Medical Center, P.O. Box 670056, Cincinnati, OH 45267 USA; 8 Department of Pediatrics and Molecular Developmental Biology, Division of Human Genetics, Cincinnati Children’s Hospital, Cincinnati, OH 45229 USA; 9 Department of Molecular Biomedical Sciences, College of Veterinary Medicine, North Carolina State University, Raleigh, NC USA; 10 Department of Ecology and Evolutionary Biology, Yale University, New Haven, CT USA

## Abstract

Following the draft sequence of the first human genome over 20 years ago, we have achieved unprecedented insights into the rules governing its evolution, often with direct translational relevance to specific diseases. However, staggering sequence complexity has also challenged the development of a more comprehensive understanding of human genome biology. In this context, interspecific genomic studies between humans and other animals have played a critical role in our efforts to decode human gene families. In this review, we focus on how the rapid surge of genome sequencing of both model and non-model organisms now provides a broader comparative framework poised to empower novel discoveries. We begin with a general overview of how comparative approaches are essential for understanding gene family evolution in the human genome, followed by a discussion of analyses of gene expression. We show how homology can provide insights into the genes and gene families associated with immune response, cancer biology, vision, chemosensation, and metabolism, by revealing similarity in processes among distant species. We then explain methodological tools that provide critical advances and show the limitations of common approaches. We conclude with a discussion of how these investigations position us to gain fundamental insights into the evolution of gene families among living organisms in general. We hope that our review catalyzes additional excitement and research on the emerging field of comparative genomics, while aiding the placement of the human genome into its existentially evolutionary context.

## Introduction

Human genomes contain groups of genes that share common ancestry and are often functionally related. These *gene families* comprise the largest proportion of the protein-coding sequences, and in many cases, they are the product of gene duplications over time. However, characterizing the sequence and functional diversity of gene families among species has been challenging. Organisms can show sequence divergence among duplicate genes, as well as differences in numbers of genes within each gene family; these variations can be observed among closely related species or even at the population level (i.e*.*, copy number variation). This molecular diversity is the manifestation of a complex evolutionary history of gene duplication over time that can result in two or more paralogs on the same chromosome (i.e*., cis*) that are either clustered or at distant chromosomal locations. Alternatively, paralogs can occur on different chromosomes (i.e., *trans*) or reflect a combination of these locations. Because many of these duplications date to deep evolutionary divergences, efforts to decode human gene families rely on interspecific comparative genomic studies between humans and other animals [1–4].

Early research comparing the genomes of model species (i.e*.*, fruit flies, mice, etc.) to humans has yielded tremendous benefits to our understanding of human genomics. This process has been markedly enhanced by the recent proliferation of genomic sequence, annotation, and transcriptomic resources for non-model species [5]. This renaissance of comparative approaches is rapidly linking human gene families and their respective functions, to a wide variety of species that previously lacked genomic resources. This expansion on the genetic resources available for non-model species has also revealed gene families that are absent in humans, but that have analogous functions in the latter. By understanding how these gene families evolve, we can reveal the processes, synergies, and constraints governing genome evolution across the Tree of Life, while providing a better understanding of the gene families in the human genome. In conjunction, these insights are crucial for informing and empowering experimental research in years to come.

In this review, we reflect on the growing body of knowledge associated with gene family evolution. We begin by highlighting the parallels between comparative genomics and phylogenomics research. We then review cases in which comparative genomic approaches have advanced our understanding of how gene families contribute to the evolution of gene expression, as well as the roles that different gene families play in the interactions of species with their biotic and abiotic environments. Next, we survey recent methodological advances and challenges and conclude by highlighting emergent research frontiers and the limits that persist for this type of research. Comparative genomic studies will continue to grow in number in parallel with the growth of high-quality genomic resources. We argue that we will be able to not only characterize gene family diversity but also decipher meaningful functional implications from variation in gene duplication, thereby quantitatively measuring the specific processes that underlie gene family evolution.

## Placing the human genome into context

The current reference assembly of the human genome, GRCh38, constitutes ~ 3100 megabases that are predicted to encode up to 20,000 protein-coding genes, over 25,000 non-protein-coding genes, and an astonishing ~ 15,000 pseudogenes (i.e*.*, non-functional copies of protein-coding genes). Around three-quarters of human protein-coding genes have at least one recognizable paralog, i.e*.*, a homologous gene that arose following duplication of an ancestral gene. This high frequency of paralogous genes indicates that much of the coding human genome can be grouped into gene families (i.e*.*, sets of homologous genes). Because of the deep ancestry of gene duplication and divergence, many of the gene families found in humans are either homologous to, or analogous with, the diversity of genes found across the Tree of Life. Consequently, the biology of human gene families can be illuminated both by their study in humans and by their comparison with other organisms. Phylogenetics provides the inferential machinery to illuminate evolution across these macro- and microscales, by informing the relation of species trees to the organismal evolution of species and the relation of gene trees to the molecular evolution of genes. Correspondingly, application of phylogenetics to our evolutionary history has shed light on the origins of gene families within *Homo sapiens.*

Phylogenetic approaches can play an important role in revealing the processes that underlie the origins and function of human gene families. Intuitively, gene family evolution is expected to reflect the evolutionary relationships among species, with divergences arising as a result of selective pressures, mutations and drift across hundreds of millions of years of evolution [6]. However, this gradual and continuous evolutionary change is not always manifest. Some gene families are more labile and can actively generate novel sequence and functional diversity, while others are highly constrained. The latter represent crucial components of the genetic architecture within the Tree of Life that constitute a historical archive of sequence and functional diversity. Though representing a conceptually useful dichotomy, these two extremes are not mutually exclusive. We can expect some gene families with tightly conserved functions to also contribute novel paralog templates that facilitate sequence diversity and innovation. Just as the inference of species trees enables us to understand functional parallels and divergences at the organismal level (i.e., macroscale), inference of gene family trees across multiple species enables us to understand functional parallels and divergences at the gene level (i.e., microscale). The rapid accumulation of genome sequences for lineages across the Tree of Life now empowers such inferences.

## The evolution of gene expression

Some of the first insights into the evolution of gene expression came from the study of gene families. Expression of gene duplicates has been shown to evolve at as much as a tenfold higher rate immediately following duplication, thereafter slowing, and to do so asymmetrically and leading to increasing gene network complexity [7]. The evolution of gene expression is known to be associated with complex gene regulatory networks (GRNs), and divergence in the former often produces changes in the latter [8]. Overall, changes in GRNs and their accompanying differences in expression play an essential role in the phenotypic diversity observed throughout the Tree of Life, making these studies highly relevant for genetics, physiology, morphology, and even ecology. Despite this clear relevance for the observed diversity, there are many challenges for directly comparing gene expression among multiple species. First, differential gene expression is expected to change significantly with genetic distance [9, 10]. Even when the relationship is not directly proportional, studies have shown that differential gene expression between even distant populations of the same species can be elevated even under “Control” experimental conditions [9]. Second, the expression dosage level of a duplicated gene is expected to be twofold; however, functional genomics experiments have demonstrated that gene duplicates can be expressed at over 2–5 times the levels of single-copy genes [11]. Third, there are many logistical issues for conducting common-garden experiments with multiple species. In addition to ethical considerations concerning human and non-human research subjects, the need to account for tissue homologies (e.g., quantitative differences in tissue content of organs of divergent species) is another challenge that arises. This challenge renders the practice of comparing expression of related genes within a single species easier than comparisons of the expression of related genes among divergent species. Newly emergent computational tools coupled with phylogenetic approaches are now allowing us to overcome such challenges. For example, a phylogenetic analysis of expression variance and evolution (EVE) is able to provide information on differences in gene expression promoted by plasticity relative to those associated with genetic divergence [12]. We are now positioned to test fundamental hypotheses concerning the evolution of gene expression [13].

Studies have suggested that gene expression evolves mostly through stabilizing selection [14], with an apparent paucity of directional selection. Estimates indicate expression divergence is usually below expectations from random mutations and genetic drift between even highly divergent species such as fruit flies, mice, and primates [15], a result often attributed to compensatory effects between *cis*- and *trans*-regulatory elements of expression [16]. Correspondingly, investigations have revealed lower differences in expression than expected between closely related species [17]. In contrast, uncommon cases of directional selection caused by mutations in coding sequences, changes in gene regulatory regions, and gene duplications are responsible for large effects on phenotype and function among divergent species [8, 18, 19]. Continued comparative investigations on the evolution of gene expression represents a fruitful avenue that could illuminate mechanisms underlying rapid phenotypic diversification.

## The impact of environmental change on genomic evolution

All organisms respond in some measure to changes in external abiotic conditions. Due to the fast pace of changes promoted by anthropogenic activities, the study of how abiotic fluctuations impact gene expression has increased dramatically in the past decades. Thus, comparative studies of gene expression between species have emerged as a critical basis for understanding how organisms respond to environmental changes. Despite the intrinsic differences between metabolic regulators and conformers, studies have found overlap in the functions that can be activated in the face of environmental stress. In particular, a great deal of overlap has been detected in mechanisms associated with metabolic compensation, as well as responses to cellular stress. These insights now position us to better predict potential genomic consequences to specific environmental changes.

How lineages respond to temperature increase has become an area of increased research focus, where there is overlap of implications between humans and other species. In the particular case of aquatic poikilotherms, increasing temperatures lead to an increase in the metabolic activity, which in turn leads to an elevated demand for oxygen consumption [20, 21]. This increase in oxygen consumption as a function of aerobic demand can lead to enhanced cellular stress due to the elevation of reactive oxygen species, which can trigger a cascade of detrimental effects for cells. In the liver, for example, responses of fishes to elevated temperature have been associated with the activation of gene categories related to the mTOR complex, oxidation–reduction, cellular apoptosis, mitochondrial electron transport chain, permeability of the endoplasmic reticulum, etc., which are also extensively reported to be activated in humans and mice exposed to intense exercise [22–24]. Similar observations have been reported in fish brains exposed to elevated temperatures, where activation of compensatory mechanisms can be similar to those observed in humans with cerebrovascular strokes or other neuro-motor ailments. For example, *Drosophila* flies and fish exposed to warm environments for multiple generations can exhibit compensation by improving neuro-motor connections by the activation of the gene plastin 3, which in humans and mice is a protective modifier for spinal muscular atrophy [25, 26]. Similarly, warm water conditions can lead to the activation of genes associated with low oxygen conditions in the nervous system of aquatic animals, as a result of the increased oxygen demand, and similar pathways are known to be activated in humans with strokes and circulatory deficiencies (*e.g.*, neuroglobins) [26]. Given climate forecasts for the next century, further investigation of how gene families evolve under changing abiotic conditions will be vital for identifying potential future risks to public health.

## Immune gene families martial a clustered frontline of defense against disease

**Fig. 1:**
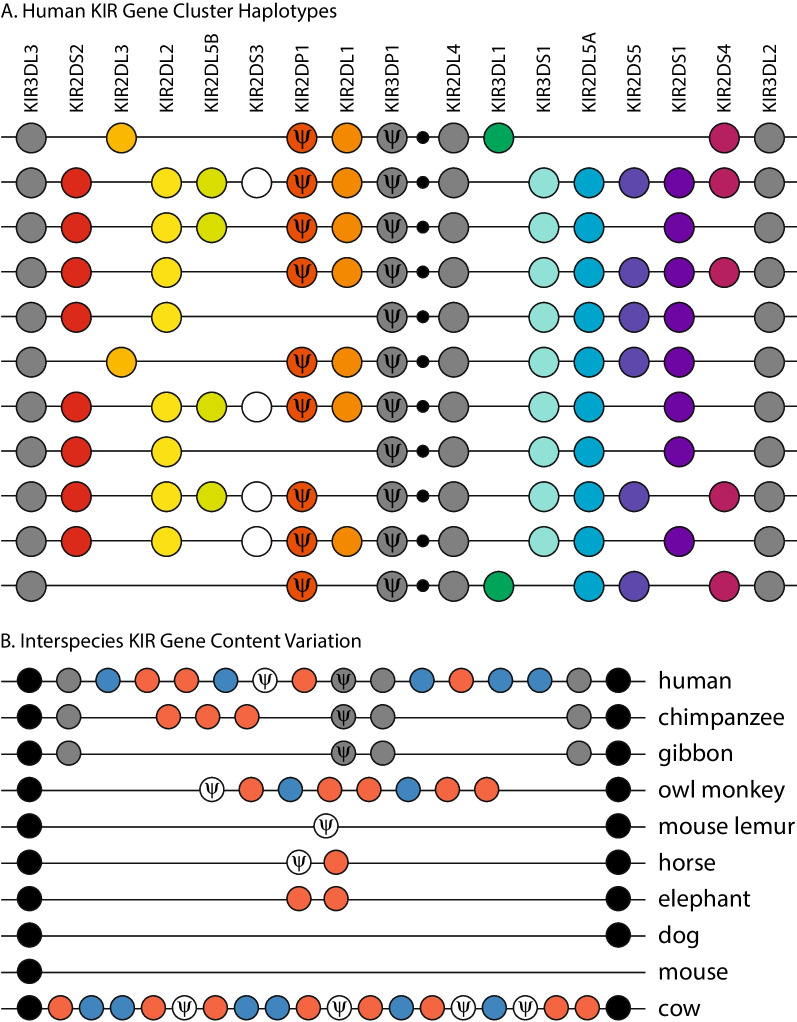
Killer cell Ig-like receptor gene clusters display dramatic gene content variation. **A** Eleven gene content haplotypes for the human killer cell Ig-like receptor cluster within the leukocyte receptor complex adapted from Middleton and Gonzelez [28]. Framework killer cell Ig-like receptor genes are conserved across haplotypes (*gray circles*), whereas other genes (*color-coded circles*) are variably present across haplotypes. Additional haplotypic variation is achieved through a recombination hotspot between *KIR3DP1* and *KIR2DL4 *[29] (*small black circle*). **B** Variation in the number and combination of killer cell Ig-like receptor genes within the leukocyte receptor complex in mammalian genomes [37–40]. Framework killer cell Ig-like receptor genes are conserved in primates (*gray circles*) and bounded by conserved flanking genes (*black circles*). The numbers of killer cell Ig-like receptors with predicted inhibitory (*red*) and activating (*blue*) functions vary between species. Additional gene content variation is observed for other gene families within the leukocyte receptor complex (e.g., leukocyte Ig-like receptors and leukocyte-associated Ig-like receptors). The figure is not to drawn scale; it is designed to highlight common sequences (*ψ*: pseudogene)

Many innate immune receptors involved in differentiating between self and non-self are encoded by gene families that are organized as clusters throughout the genome and encode both activating and inhibitory forms. For such families of clustered genes, high sequence diversity and even diversity in the presence or absence of gene orthologs between humans and more distantly related vertebrates should be expected. Such a fast pace of molecular evolution is necessary to compete in the evolutionary arms race with rapidly evolving pathogenic threats [27]. However, there is also significant diversity in sequences and even genes between individual humans. For instance, the leukocyte receptor complex (LRC) is likely one of the best studied examples of intraspecific gene content variation, in which different people encode different numbers and combinations of killer cell immunoglobulin-like receptor (KIR) genes which play important roles in natural killer (NK) cell function [28] (Fig. 1A). A large-scale study that defined the KIR haplotypes for 800 families of European origin resolved more than 3000 haplotypes and identified over 70 unique haplotypes based solely on gene content [29]. Different KIR gene content haplotypes have been linked to differences in susceptibility to infectious diseases such as HIV and malaria [30–32] as well as autoimmune disorders [33] and are important factors for the success of hematopoietic stem cell transplantation for leukemia patients [34].

**Fig. 2:**
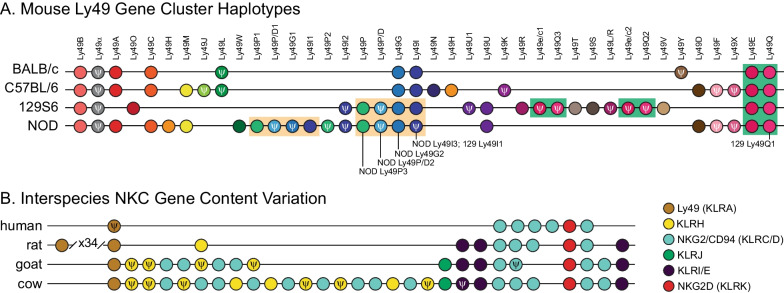
Ly49 and the NKC gene clusters display dramatic gene content variation. **A** Ly49 haplotypes for four mouse strains adapted from [48] showing genes with sequence homology (*color-coded circles*) and sets of genes that likely arose through recent duplication events (*shaded rectangles*). The Ly49 nomenclature as opposed to the standardized Klra# gene nomenclature has been used as some of the genes listed are not in the mouse reference genome and hence do not yet have approved gene symbols. **B** Gene content variation of Ly49 (KLRA; *brown*), KLRH (*yellow*), NKG2/CD94 (KLRC/D; *blue*), KLRJ (*green*), KLRI/E (*purple*), and NKG2D (KLRK; *red*) genes in the NKC adapted from [293]. The figure is not drawn to scale; it is designed to highlight common sequences (*ψ*: pseudogene)

Clustered families of innate immune receptors are not unique to humans. However, expansions and contractions of these gene families vary substantially across the Tree of Life. For example, mice do not have an expanded cluster of KIRs. Instead, gene family variation similar to human KIRs is evident in the natural killer complex (NKC) of mice, in which different inbred mouse strains encode different numbers and combinations of Ly49 genes, also known as killer cell lectin-like receptors or Klras. In rodents, these genes play similar roles in NK cells as KIRs do in humans [35] (Fig. 2A). The diversity of Ly49/Klra gene cluster content in mice stands in contrast to the single *KLRA1P* pseudogene in the human genome and is linked to differences in susceptibility to murine cytomegalovirus (MCMV) infection [36]. Given the intraspecific gene content variation that is observed for numerous gene families in humans (KIRs) and mice (Ly49/Klra), this discrepancy challenges us to consider which vertebrate lineages possess gene family expansions convergent with, or functionally analogous to human expansions. Moreover, a comparative perspective enables us to derive general rules that underlie the diversification of clustered vertebrate immune genes and the maintenance of specific levels of intraspecific variation.

In the case of the LRC, KIRs display dramatic convergences in gene content variation between species. Humans encode 4–20 KIR genes (including rare haplotypes [29]) and cows encode up to 18 KIR genes, whereas multiple other vertebrates encode one or two KIRs, or, like mice, have lost all KIR genes at the LRC (Table 1) [1, 37–40] (Fig. 1B). However, studies on the *intraspecific* gene content variation of the LRC are limited primarily to KIRs in primates and very little is known about this level of structural variation in other mammalian lineages. Further, the LRC contains multiple gene families that encode innate immune receptors structurally similar to KIRs, such as the leukocyte Ig-like receptors (LILRs) and leukocyte-associated Ig-like receptors (LAIRs) [41, 42]. These LRC loci have been associated with multiple immunological disorders including rheumatoid arthritis, multiple sclerosis, and lupus [43], are expressed on a range of immune cell types, and have been classified as activating or inhibitory based. Moreover, the LILR genes in the LRC also display interspecific gene content variation. Respectively, reference genomes for humans, cows, horses, and elephants encode 11, 26, 3, and 2 orthologs of LILR genes [38, 39, 44] with mice also encoding at least 8 LILR orthologs (also referred to as paired Ig-like receptors or PIRs) [45, 46]. Even among LAIRs—for which humans encode only two genes (*LAIR1* and *LAIR2*)—three LAIR-like sequences have been reported in pig [47] and elephant [38], and Ensembl predicts that a number of mammalian species encode paralogs of *LAIR1* (e.g., black snub-nosed monkey, vervet, drill, greater horseshoe bat) or have lost *LAIR1* (e.g., polar bear, armadillo, shrew, dolphin).

**Table 1:** Interspecific gene content variation of the KIR gene family

Species	# KIR genes in LRC	References
Homo sapiens	4–20	[29]
Chimpanzee	5–11	[281]
Orangutan	5–10	[282]
Gibbon	2–5	[283]
Baboon	> 6	[284]
Macaque	4–17	[285]
Owl monkey	8	[286]
Gray mouse lemur	1	[287]
Mouse	0	[288]
Rat	1	[288]
Pig	1	[289]
Cow	18	[40]
Horse	1	[39]
Elephant	4	[38]
Dog	0	[49]
Cat	1 pseudogene	[49]
Sea lion and seal	1	[49]

Expansions of clustered gene families such as these LRC loci are predicted to increase receptor diversity, an increase that can enable a better defense against the next unknown pathogen. Why these lineage-specific expansions are heterogeneously distributed across the vertebrate Tree of Life remains less clear. A potential explanation may lie in functional overlap. For example, KIR and Ly49/Klra proteins are considered functional analogs, as both interact with MHC class I proteins, mediating the recognition and direct killing of infected and cancerous cells. The NKC gene cluster, which includes the Ly49/Klra genes, displays dramatic interspecific gene-content diversity (Fig. 2B). There is a single Ly49/Klra locus in humans and dogs, compared to 6 and up to 21 Ly49/Klra genes in horses and mice, respectively (Table 2) [39, 48, 49].

**Table 2:** Interspecific gene content variation of the KLRA (Ly49) gene family

Species	# Ly49 genes in NKC	References
Homo sapiens	1 pseudogene	[290]
Chimpanzee	1 pseudogene	[284]
Orangutan	1	[291]
Gibbon	1	[284]
Baboon	1	[284]
African green monkey	1	[284]
Gray mouse lemur	1	[287]
Mouse	8–21	[35]
Rat	33–36	[35]
Pig	1	[292]
Cow	1	[293]
Horse	6	[294]
Dog	1	[49]
Cat	1	[292]
Sea lion and seal	1	[49]

As divergences between humans and other vertebrates span deeper timescales, finding functional analogs becomes increasingly challenging. Initial studies looking to identify mouse KIRs led to conflicting results [50, 51]. We now know that KIRs and Ly49/Klras are structurally different proteins and do not share a common genetic origin, but play comparable roles in NK cells’ surveillance for transformed and infected cells. As mentioned above, humans encode a single Ly49/Klra pseudogene *(KLRA1P)* and mice have lost the KIR genes from the LRC. This extreme case of convergent evolution highlights what can be missed when only two species are compared. Initial hypotheses, prior to the completion of the sequencing of the human genome, was that primates used an expanded set of KIR genes and rodents used an expanded set of Ly49/Klra genes for the same NK function and that an expansion of the KIR or Ly49/Klra genes was required for a functionally competent immune system. As genomic sequencing became more affordable and the LRC and NKC were sequenced from a wider range of mammals, it became obvious that certain lineages expanded the KIR cluster (e.g., primates and cattle), while others expanded the Ly49/Klra cluster (rodents and equidae), and yet others encode a single KIR and a single Ly49/Klra gene (e.g., pinnipeds) [49]. This observation in seals and sea lions made it clear that the long-term survival of placental mammals does not require an expanded system of either Ly49/Klra or KIR receptors.

Natural killer (NK) cells have been described as one of the oldest immune cell types that differentiates between self and non-self. Although NK cells have been functionally described from bony fish [52, 53], numerous efforts to identify genes encoding KIRs or Ly49/Klra in fish have been unsuccessful [1]. Nevertheless, in the same way that humans use KIRs and mice use Ly49/Klras to mediate NK function, a candidate gene family that may facilitate mammalian-like NK cell function in fishes is the novel immune-type receptors (NITRs) gene family [54–57]. Similar to mammalian KIRs, NITRs are encoded in gene clusters, include inhibitory and activating forms, and display gene content variation [1, 55, 58]. NITRs have recently been found to have ancient evolutionary origins within the earliest divergences of ray-finned fishes, an evolutionary persistence that lends support to the hypothesis that this receptor family offers some core immune response functionality [59–62]. Unfortunately, it is not known if NITR evolution is analogous to that of KIRs and Ly49/Klras. This lack of understanding imposes limits on our ability to link NK cell function in teleost model organisms (e.g., zebrafish, stickleback, and medaka) to the mammalian immune system. Because there are numerous immune receptor families that display both inter- and intraspecific gene-content variation across ray-finned fishes and are not identifiable in mammalian (or other tetrapod) genomes [59, 60, 63–65], comparative genomic investigations of the molecular basis of self-recognition pathways in “the other half” of all living vertebrates are an exceptionally rich research frontier that can aid us in understanding the evolution of our own genomes.

## Molecular biology and its translation to action against cancer

Genomic analyses of human cancers have revealed several phenotypes that are shared across a vast majority of types and have been dubbed “cancer hallmarks” [66, 67]. These hallmarks include immune evasion, resisting cell death, and evasion of growth suppressors to name but a few. In many cases, the genetic basis of these and other cancer hallmarks can be linked to gene families that are broadly distributed among mammals or other vertebrates. For example, the TP53 (p53) gene family arguably represents the most well-studied family of human oncogenes [68]. This gene family has evolutionary origins dating back to the most recent common ancestor of choanoflagellates and metazoans, where a single gene involved in germline DNA repair has been maintained [69]. This gene duplicated multiple times during the evolution of jawed vertebrates, giving rise to *TP53*, *TP63*, and *TP73*. While decades of comparative research have defined general features of this gene family’s evolution and biology [68], recent comparative studies have illuminated the role of this gene family in mitigating the risk of cancer development in the largest land mammals—elephants.

Across vertebrates, there is a positive relationship between the risk of developing cancers and increases in body size [70]. This relationship is a consequence of a larger cell count increasing the chance of malignant cell transformations [71]. Additionally, the risk of developing cancer is positively correlated with longevity [72]. As a consequence of their large-body size and long life spans, elephants should be expected to have some of the highest rates of cancer among terrestrial mammals. However, this expectation is contradicted by empirical data. Elephants achieve a seemingly paradoxical low rate of cancer incidence in part through a duplication of TP53 genes that provide a greater sensitivity to DNA damage [68]. Recent studies have shed light on genes interacting with elephant TP53, revealing that the *LIF* (leukemia inhibitory factor) gene has undergone segmental duplication in selected species including the African elephant. Across analyzed genomes, most copies of this gene are pseudogenized. Elephants stand out as an exception, with investigators finding one gene (*LIF6*) that had refunctionalized with apoptotic function [73]. These studies and others like them have created an exciting opportunity to investigate the genetic basis of DNA damage repair mechanisms.

Beyond the genetics of DNA repair mechanisms, it is also clear that studies of other oncogenes between species can inform the development of new therapies. To effectively guide research in cancer biology, it is important to place these oncogenes into a phylogenetic context. For example, the ALK receptor tyrosine kinase gene (previously “anaplastic lymphoma kinase”) (*ALK*), a prominent receptor tyrosine kinase (RTK) proto-oncogene, is present across a range of metazoans that spans humans to fruit flies with a high degree of structural conservation [74, 75]. *ALK* has been associated with tumorigenesis in neuroblastoma, non-small cell lung cancer, anaplastic large-cell lymphoma, breast and renal cell carcinomas and identified as a potential target for therapeutic development [76–78]. Although studies of model organisms have revealed fundamental insights into the biology of oncogenic alterations, linking these heterogeneous insights to human cancers was challenged by a lack of a comparative framework. It has recently been demonstrated that in the early history of jawed vertebrates, an ancestral *ALK* duplicated to give rise to the leukocyte receptor tyrosine kinase (*LTK*) gene, and that subsequently *ALK* and *LTK* have traded functional roles between ray-finned fishes and sarcopterygians (e.g., tetrapods, lungfish, and coelacanth). Additionally, even as the homology among *ALK*-like genes becomes increasingly clear, the ligands for ALK in non-vertebrates {Jelly belly (*Jeb*) in *Drosophila melanogaster* [79], hesitation behavior-1 (*hen-1*) in *Caenorhabditis elegans* [80], and *ALKALI/2* (augmentor/FAM150) in humans [81, 82]} are not similarly homologous [74]. This example highlights the critical need for an evolutionary perspective in model organism-based oncogene research to effectively translate findings regarding homologous genes and their interacting partners.

Studies of oncogenes have been crucial in revealing how cancer co-opts existing gene families to enable its persistence. For instance, studies of the genetic mechanisms of tumor growth or DNA repair remain invaluable. However, an additional frontier in cancer biology leverages comparative immunogenetics to investigate the evolution of receptors that modulate the adaptive and immune system response, to better understand how cancers evade detection or elimination. An example of how cancer co-opts the genetic machinery of the mammalian immune system has been revealed by studies of signal regulatory proteins (SIRPs), a family of transmembrane glycoproteins with extracellular immunoglobulin-like domains, involved in the regulation of tyrosine kinase-coupled signaling processes [83, 84]. SIRPs have been identified broadly among mammals including humans, primates, rodents, dogs, cows, horses, and opossum [85, 86]. In humans, the SIRP family contains the inhibitory SIRPα, activating SIRPβ, non-signaling SIRPγ, and soluble SIRPδ encoded in a gene cluster on chromosome 20 [83, 86, 87]. Putative clusters with different numbers of SIRP homologs have been observed in other mammals, birds (*Gallus*), and lizards (*Anolis*) [85, 86, 88]. The evolutionary origins of SIRPs remain unknown, as does the scope of predicted functional conservation.

Multiple cancer cells utilize the relationship between SIRPα and its ligand CD47 to prevent tumor cell phagocytosis [89, 90]. CD47 is commonly overexpressed on the cell surface of many cancers, providing a “Don't eat me” signal that engages SIRPα on macrophages and prevents phagocytosis [91, 92]. SIRPα recognizes the elevated levels of CD47 on tumor cells and negatively controls effector function which prevents destruction of the cancer cells [93, 94]. However, experimental administration of monoclonal antibodies or soluble SIRPα (Fc-fusion) that bind CD47 and block the CD47-SIRPα signaling pathway promotes tumor cell phagocytosis, inhibits tumor growth in mice, and increases survival [92, 95]. A large-scale comparative perspective of immune gene families such as SIRPs and their interacting partners is therefore of high value for the design of new therapies and for revealing natural systems with high translational relevance.

## Comparative genomics illuminates the ancestry and diversity of vision

Light sensing is one of the most ancient characteristics of life [96], and proteins associated with human vision have been studied for over a century in various model organisms [97, 98]. These loci have been used in studies that span investigations of molecular evolutionary rates [99], the reconstruction of ancestral proteins in extinct species [100], dim-light vision [101, 102], and extensive applications for molecular systematics [103–105]. However, it has only been in the last few decades that large comparative studies have begun placing gene families in the human genome into evolutionary context. The results of these studies have revealed numerous associations between gene family evolution and the ecology of a lineage, as well as a remarkable scope of genotypic diversity across the Tree of Life associated with light sensing phenotypes.

Families of photoreceptors are widely distributed across multiple phyla—including plants and fungi—that rely on those sensory pathways to regulate their responses to environmental changes [106, 107]. Although these lineages do not possess any structures analogous to human eyes, their diversity of light sensor molecules is extraordinary. Comparatively, fungi have a greater diversity of light sensor molecules than humans, each providing sensation of different ranges of ambient light. In addition to retinal binding rhodopsin (like humans) for sensing green light, fungi also feature phytochrome- and flavin-based photoreceptor gene families, providing red- and blue-light sensation [108]. Gene duplications of fungal photoreceptors are present across many species [108], and their expansion has been clearly linked to fungal ecology [106, 109, 110]. For example, duplication and divergence in fungal rhodopsins and opsin-like proteins have been characteristic of clades of pathogenic and non-pathogenic fungal species [111], whereas extreme expansion of the phytochrome gene family has been found in aquatic fungi with multiple paralogous copies [112].

Among gene families associated with human vision, crystallins represent perhaps one of the most iconic examples of an evolutionary bloom with hypothesized lineage-specific gains and losses that are associated with the ecology of the vertebrate lens [113–115]. Numerous reviews and in-depth studies of human crystallin gene families have been conducted due to their essential role in the maintenance of lens clarity, and variation in human crystallin genes has been linked to cataracts and to vision loss [113, 116–118]. However, the evolutionary history of *γ*-crystallin genes has only recently come to light and this gene family represents an intriguing empirical example of a gene family considered “on its way to extinction” in humans [119]. A diverse suite of *γ*-crystallins associated with the generation of a high refractive index are found in species that possess a hard lens with low water content such as fishes [113, 119]. In contrast, convergent losses or loss of function of *γ*-crystallins have been reported in lineages such as primates or birds that are characterized by soft lenses with high water content [113, 119]. Humans are estimated to have lost function in two-thirds of the *γ*-crystallins found in some rodents [120]. Therefore, crystallin gene families are particularly well positioned for future studies investigating the evolution of gene loss.

In addition to crystallins, opsins represent another spectacular example of an evolutionary bloom. Since the sequencing of the first opsin gene nearly 40 years ago in bovids [121, 122], over 1000 opsins have been identified in many species including human, fly, mouse, and zebrafish [123]. Detailed examination of these genes has revealed the basis of color vision across the Tree of Life, often demonstrating striking cases of functional convergences [124, 125]. For example, there are correlations between the opsin repertoire and ecological factors [126, 127], gene losses or loss of function associated with trade-offs between sensory systems [128], amino acid convergence among distantly related taxa (i.e*.,* fish; [129–131]), and convergences in the genetic basis of many human retinal degenerative diseases and vision disorders [132, 133]. The diversity of opsin genes is perhaps not surprising when considering the scale of evolutionary divergences that span lineages such as dragonflies, ray-finned fishes, and humans [134, 135]. However, that this diversity of opsin genes across metazoans is expressed not only in the eye but also in the skin and peripheral tissues has only recently become appreciated [136, 137]. Research in the past decade has revealed a diversity of opsin genes expressed in the skin and other organs of animals including humans [136, 138] and made clear that opsin genes carry out functions that expand beyond light reception. An increased focus on the comparative genomics of opsin gene families already has implications for wound healing, hair growth, optogenetics, and metabolic physiology to name but a few [136, 139]. Future comparative genomic investigations are clearly poised to inform functional experiments that can illuminate the full diversity of these gene families.

## Sniffing out the genomic basis of diverse chemosensation receptors

Chemosensation is ubiquitous throughout the animal Tree of Life. However, the molecular basis of chemosensation has only recently been elucidated. Discovery of chemosensation receptor genes occurred within the last two to three decades, and its discoverers were awarded a Nobel Prize in 2004 [140, 141]. The mechanism of chemosensation involves the conversion of environmental chemical information into neurophysiological information in the brain and is relatively similar across vertebrates and invertebrates [142, 143]. Volatile and nonvolatile chemicals interact with proteins encoded by genes of large multi-copy gene families expressed in sensory neurons and supporting cells of the olfactory and gustatory systems (*cf* [143] *for review*). Molecular investigations of odorant receptors have revealed that these account for the largest gene families in animals, encoding for up to 5% of the protein-coding genome in mammals alone [144–146]. As these gene families are among the fastest evolving in the genome [147], this makes them ideal models for understanding complex patterns of gene family evolution.

Chemosensory gene families are characterized by rapid evolutionary rates through high gene turnover and rapid diversification of homologous genes [146, 148–151]. The convergently evolved odorant receptor gene families in vertebrates and insects [146, 152, 153], for example, demonstrate some of the most extraordinary patterns of gene duplication and pseudogenization in animals, constantly expanding and contracting as species evolve through time [148]. We have made tremendous strides in our annotation and delimitation of human odorant receptors. However, placing these into the context of the vertebrate Tree of Life is challenged by their complex evolutionary history.

**Fig. 3:**
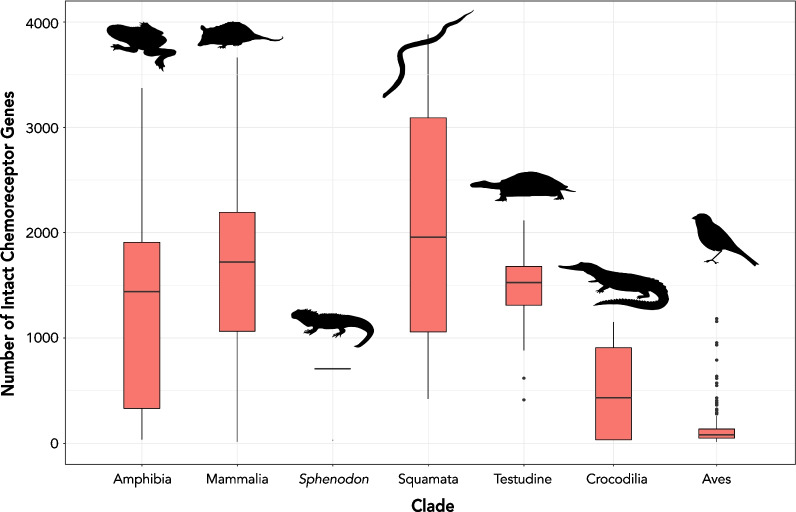
Number of intact chemoreceptors in available tetrapod genomes harvested using methods described in Yohe et al. [147]. Chemoreceptors include olfactory receptor genes (Class I and Class II), vomeronasal receptor (type 1 and type 2) genes, γ-c receptor genes, and trace amine-associated receptors. These counts include genes with > 650-bp open reading frames and do not include any pseudogenes. Silhouettes are from PhyloPic

The expansion and contraction of the odorant receptor gene family have been linked to changes in the sensory ability of some animal lineages, including the adaptation to novel food resources [148, 154–157] or specializations in the social–chemical communication system [158, 159]. Accordingly, the hundreds/thousands of copies of distinct olfactory receptors likely reflect the diversity of odorant ligands that can be detected by the organism (Fig. 3, [144, 145]). Functional assays of olfactory receptor repertoires in a few model organisms suggest that odorant receptors act as labeled lines or in a combinatorial fashion, thereby enabling the specific detection and discrimination of chemicals and chemical mixtures vastly exceeding the number of receptor genes encoded in the genome, while also being highly specific [160–167]. These observations have translational implications for understanding chemoperception at the population level, and future studies of the functional consequences of odorant receptor variation between individuals offer a particularly exciting avenue of future research.

**Fig. 4:**
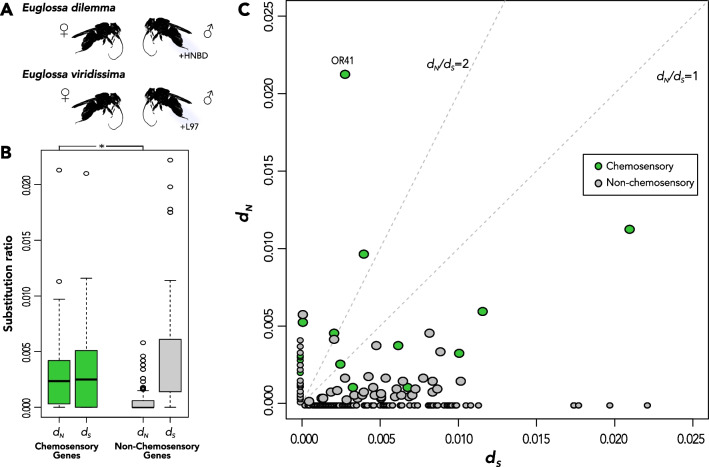
Molecular evolution of orchid bee chemosensory receptors. **A** Males of sibling *Euglossa* species [335] manufacture perfumes to attract females and differ by a single compound per species (noted as + HNBD/+ L97) in this system [180, 336–338]. **B** Rates of nonsynonymous substitutions (*d*_*N*_) in *Euglossa* chemosensory genes are significantly higher (denoted with *asterisk*) than in non-chemosensory genes. **C** *d*_*N*_ versus rates of synonymous substitutions (*d*_*S*_). Selection analyses reveal candidate chemosensory receptors (*e.g., Or41*) under divergent selection in the two sister species, potentially related to perfume differences. Figure adapted from Brand et al. [185] under a Creative Commons Attribution 4.0 International License (http://creativecommons.org/licenses/by/4.0/). If an adaptive hypothesis is maintained, it is expected that the species divergent in OR41 might bind to + L97 in *E. viridissima* and + HNBD in *E. dilemma*, but this binding has yet to be experimentally demonstrated

The sheer enormity of these receptor gene repertoires (Fig. 3) calls into question the functional purpose of the extensive redundancy of these genes. Studies have repeatedly shown the elevated rates of evolution of chemoreceptor genes across animals, insects, and vertebrates alike [147, 155, 168]. Adaptive scenarios predict elevated rates of nonsynonymous substitutions over short lengths of evolutionary time (i.e*.*, relative to rates of neutral synonymous substitutions) and would likely correspond to an environmental chemical signal or signals relevant to fitness. Evidence for adaptation is prevalent in *Drosophila*, where behavioral and biochemical assays have repeatedly demonstrated specialization of odorant genes in *Drosophila* species to ligands of host fruits (e.g., *Drosophila sechellia* and the host shift to morinda fruit) [169–173]. However, outside of model organisms, interpretations of correlative patterns have emerged but are still inconclusive. For example, orchid bee (Apidae: Euglossini) males collect specific organic chemical compounds from floral and non-floral sources available in their environment to create a perfume [174–176] released during a stereotypical display behavior at perching sites, and it is only in combination with perfume display that mating occurs [177–182]. Perfumes are most likely involved in sexual selection, presumably by enabling species-specific recognition or as an indicator of male fitness (Fig. 4A, [183, 184]). Homologous chemosensory receptor genes are highly differentiated and evolve under strong divergent selective pressures between even recently diverged (~ 150 kya) orchid bee lineages (Fig. 4B, C), suggesting adaptive differentiation of chemosensory genes [185, 186]. Although there is some evidence that divergence in perfume signals might be correlated with divergence in chemosensory genes [185], the link between genotypic and phenotypic evolution is still missing.

The scarcity of evidence of strong selection for direct receptor-odorant ligand binding suggests that the relationship between chemoreceptor genes and the environment is more complex than initially hypothesized. Rates of nonsynonymous substitutions for dozens of genes are consistent with diversifying selection across tetrapods [147, 168]. Substitution rates in the mouse lemur (*Microcebus*) pheromone receptors (vomeronasal type 1) are so high, and they occur at such exceptional copy numbers, that they have been described as genes on the verge of a “functional breakdown” [187]. Is it really possible that these dozens of chemoreceptor genes are solely under extensive positive selection? Neotropical bats illustrate an instance where differences in molecular rates are not directly explained by dietary ecology (e.g., plant-visiting versus animal feeding), but rather are associated with the chromosomal location of the genes [188]. Even within *Drosophila*, chemosensory genes have an elevated background of standing variation that may facilitate rapid adaptation in the event of shifts in ecological niches [189].

**Fig. 5:**
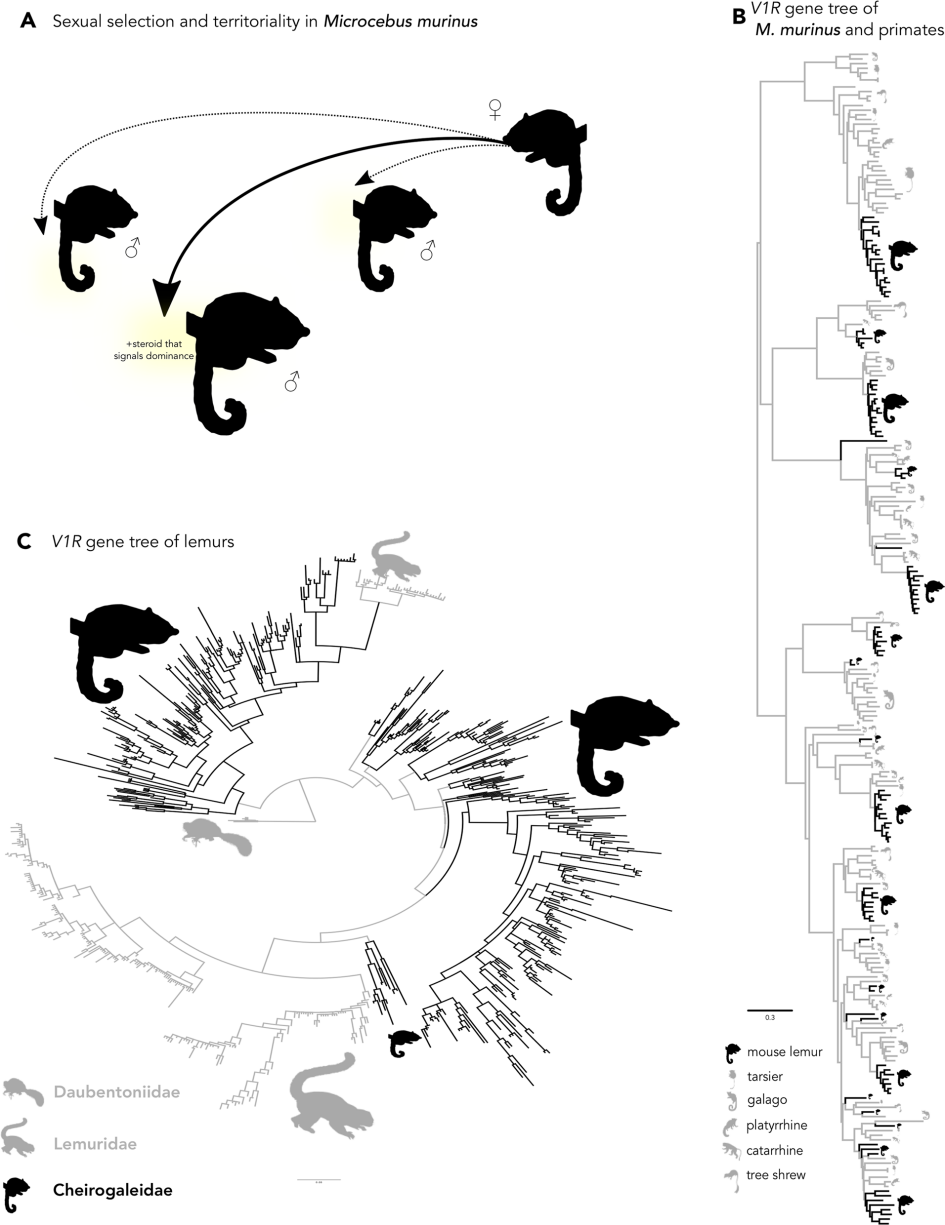
Vomerolfactory-mediated courtship and territoriality in mouse lemurs and the phylogenetic history of vomeronasal receptor type 1 (V1R) genes in primates. **A** The urine of the dominant male gray mouse lemur (*Microcebus murinus*) often contains a distinct steroid-like compound that suppresses reproductive behavior of other males, but it must stand out among competitors to attract females. *Dotted-lined arrows* indicate a weaker signal among the dominant male urine signal. **B** Gene tree of V1Rs in primates [187, 191], including the gray mouse lemur. *Black branches* indicate genes belonging to the mouse lemur, while *gray branches* belong to other primate groups. **C** V1R gene tree of lemurs [187], including several species of mouse and dwarf lemurs (Cheirogaleidae). *Black branches* are Cheirogaleidae and *gray branches* are other strepsirrhine primates. Sexual selection coupled with extensive gene duplication of vomeronasal receptors may have facilitated rapid speciation in Cheirogaleidae. Silhouettes were obtained from PhyloPic

What is the evolutionary explanation of maintaining extensive copy numbers of highly variable but seemingly redundant genes in the genome? The answer may lie in navigating a complex world incorporating a highly dynamic chemical space [151, 190], as illustrated by mouse lemurs (*Microcebus*). In contrast to other primates (including humans) that typically possess few copies of pheromone receptor genes, these largely solitary primates possess orders of magnitude higher copy numbers of pheromone receptors (Fig. 5, [191]), and the primary form of conspecific communication in these arboreal and nocturnal primates occurs via pheromonal cues of scent-marking and anogenital dragging [192–194]. Females in estrous induce sexual behavior in males through proteins in their urine, and, in response, males establish their territories via urine scent-marking to signal dominance (Fig. 5A, [192, 195]). The high rates of molecular evolution of chemoreceptors [187, 196] and rapid turnover of genes may facilitate sexual selection and species-specific adaptation [151] in this species. However, this adaptation does not come in the form of selection for a single variant binding to a specific ligand [187]. Rather, evolution of new receptors via gene duplication provides a genomic substrate to detect novel chemical cues signaled by competing males. Pseudogenization of receptors no longer relevant to the present environment leads to very low levels of orthology among closely related species (Fig. 5B, [197–199]). Mouse lemurs have speciated rapidly [200, 201]. Because receptors have continued to diversify among closely related species (Fig. 5C), it is a plausible hypothesis that increased redundancy and sequence variation among duplicates facilitate species diversification. This pattern is in sharp contrast to what is observed in bat vomeronasal receptors, in which there are very few copy number variants and a high retention of orthologs since bats diverged from ungulates and carnivores [202]. When these large gene families show low rates of gene retention at deep time scales, this may hint at conserved innate function.

Extrapolating function and adaptation across species, in particular for receptors, remains challenging. Identifying orthologous receptors among a pool of species- and individual-specific paralogs is not straightforward. Orthologs are usually assumed to have similar functions across species. However, orthologous olfactory receptors do not consistently share function [203]. In some cases, the same olfactory receptor in two species will respond to the same odorant ligand. In other cases, orthologs may differ in their responses to the same stimuli. Nevertheless, detecting adaptation in olfactory receptors is tractable with well-parameterized models of molecular evolution and appropriate consideration of protein structure and function [204]. These receptors directly interact with environmental cues, with essential roles in the identification of stimuli that historically were directly related to fitness (e.g., finding food, mates, and predator avoidance). Comparative phylogenetic approaches will be critical for understanding this nexus of complex sensory-signaling and perception [190]. Given the immense representation of chemoreceptor genes in the human genome, understanding the complex evolutionary dynamics of these genes is crucial to understanding ourselves.

## Breaking down the basis of enzymic metabolism

Enzymic metabolism genes have been speculated to evolve upon exposure to specific novel environmental pressures, including the ingestion of plants, fungi, lower eukaryotes, and bacteria. The importance of cytochrome P450 (CYP) genes in “animal–plant warfare” was emphasized long ago [205], and the CYP2, CYP3, and CYP4 families encode enzymes that participate not only in metabolism of important endogenous substrates, but also in the metabolism of plant–fungal–bacterial–viral metabolites, drugs and other environmental pollutants, pigments, and biosynthesis of pheromones. Therefore, evolution and expansions of these gene families are extremely sensitive to changes in the environment of the organism, resulting in rapid up- and downregulation of gene expression. “Evolutionary blooms” would be expected in these three human P450 families and, indeed, they have been found [206]. Evidence of these (evolutionarily recent) blooms is presented by the high dissimilarity evident when comparing human and mouse CYP genes (Table 3).

**Table 3:** Many of the heavily studied major classes of human enzymic metabolism genes

Mechanism	Encoded enzymes (gene root symbols, where applicable)	Families, classes, groups*	Representative reference(s)
*Oxidation*			
	Hydroxylases	Dozens of classes; > 1000 genes in genome	[295, 296]
	CYP monooxygenases (CYP; P450s)	Total of 18 families; 57 genes in human genome versus 102 in mouse	[206, 213, 297]
		*CYP1* (3 in *Homo*; 3 in *Mus*)	[206]
		*CYP2* (16 in *Homo*; 50 in *Mus*)	[206]
		*CYP3* (4 in *Homo*; 9 in *Mus*)	[206]
		*CYP4* (12 in *Homo*; 20 in *Mus*)	[206]
		*CYP5, 7, 8, 11, 17, 19, 20, 21, 24, 26, 27, 39, 46, 51*	[213]
	Flavin-dependent monooxygenases (FMO)	6 genes	[298, 299]
	Alcohol dehydrogenases (ADH)	8 genes	[300, 301]
	Aldehyde dehydrogenases (ALDH)	11 families, 4 subfamilies, 19 genes	[302, 303]
	Lysine (histone) demethylases (KDM)	8 groups; 24 genes	[304, 305]
	Monoamine oxidases (MAO)	Two genes	[306]
	Other amine oxidases	> 50 genes	[307]
	Peroxidases (PEX)	> 30 genes	[308, 309]
	Catalase	Three main groups; one gene in human	[310, 311]
	Oxidoreductases	< 40 genes, subunits	[312, 313]
*Reduction*			
	CYP reductive reactions (CYP)	CYP1, 2, 3, and 4 families	[314]
	NADPH-quinone oxidoreductases (NQO)	Two genes	[315]
	Aldo-keto reductases (AKR)	Two families, 7 subfamilies, 19 genes	[316]
	Carbonyl reductases (CBR)	Three genes	[316, 317]
	Short-chain dehydrogenases/reductases (SDR)	Up to 77 protein-coding genes	[318]
	Oxidoreductases	> 50	[318, 319]
*Hydrolysis*			
	Epoxide hydrolases (EPHX)	Up to 7 genes	[320, 321]
	Amidases	Three genes	[322]
	Esterases	At least 8 genes	[323, 324]
	Lipases (LIP)	22 genes	[325]
*Conjugation*			
	UDP glucuronosyltransferases (UGT)	4 families; 5 subfamilies; 23 genes†	[326, 327]
	Glutathione *S*-transferases (GST)	8 families; 21 genes	[328]
	Soluble sulfotransferases (SULT)	4 families; 9 subfamilies; 14 genes	[329]
	Membrane-bound sulfotransferases	37 genes	[330]
	*N*-acetyltransferases (NAT)	26 genes; two groups; GCN5-related (24 genes) and arylamine-related (2 genes)	[331, 332]
	Other transferases	Up to 41 groups; 265 protein-coding genes	HGNC

*Virtually all these enzyme classes, and members of each gene group with their chromosomal locations, can be found in genenames.org (HGNC)

†The “number of UGT genes” is debatable, because of the complex structures of the UGT1 and UGT2 loci

Details of the CYP gene family are perhaps the most studied on this list. The mammalian CYP2, CYP3, and CYP4 families each have many genes encoding plant-metabolizing enzymes, and the number of genes in each family varies widely among mammals [206]. The CYP1 family is involved in some plant-metabolizing enzymes but also arachidonic acid pathway-associated lipid mediator substrates [333], steroids, pigments, polycyclic aromatic hydrocarbons, polyhalogenated biphenyls, etc. The remaining 14 CYP families encode evolutionarily highly conserved enzymes with much more highly specific endogenous substrates (ablation of many or most of these is lethal), and the number of genes in each family is virtually identical among all mammalian genomes [213, 334]

The expansion of CYP genes reflects a larger phenomenon in which signals from the microbiome—which is commonly described as bacteria, fungi, viruses, and other microbes living synergistically in the digestive tract of the host [207, 208]—have had a profound effect on the evolution of host genomes. However, “digestive tract” signifies more than just “animals with stomachs”; guts have deep evolutionary origins. For example, the ambulacral groove of animals in the phylum Echinodermata or class Asteroidea and Edrioasteroidea extends from the mouth to the end of each ray or arm; each groove of each arm, in turn, has four rows of hollow tube feet that can be extended or withdrawn [209]. Even the cavitation of animals in the phylum Cnidaria (e.g., sponge, jellyfish, sea anemones and corals, etc.) functions in digestion of food and, thus, is likely to have a microbiome. Accordingly, it is likely that microbiome metabolites (as well as phytoplankton and other ancient simple plants) were among the first environmental signals to have been received by animal hosts and that their presence in turn generated selective pressure in the host to metabolize microbial metabolites.

Selective pressures on receptors of microbial metabolites—and the corresponding evolution of enzymatic genes that interact with these receptors—likely shaped major trends in genome evolution [210, 211]. Hence, what we see today are multiple transcription factors (e.g., AHR, AHRR, ARNT, NR1I3 (CAR), PPARA, PPARD (PPAR-beta), PPARG (PPAR-gamma), NFKB1/2, HNF4A/G, HNF1, NFE2L2 (Nrf2), NR1I2 (PXR), NR1H4 (FXR), ESR1/2, PGR, GHR, NPAS1, etc. [207, 212]**)**, up- and down-regulating dozens or hundreds of genes that are members of enzymic metabolism gene families. As far as the CYP gene families (Table 3), transcription factors for the CYP1, 2, 3, and 4 families involved in plant metabolite and pheromone metabolism are distinctly different from the remaining fourteen P450 gene families (CYP5, 7, 8, 11, 17, 19, 20, 21, 24, 25, 26, 27, 39, and 46) that participate almost exclusively in endogenous critical life function pathways, instead of exogenous chemical metabolism [213]. This difference in function has opened new research opportunities. For example, mice having the ablation of all Cyp2c genes in a cluster are completely viable, except for changes in metabolism for specific drugs or chemicals. As such, there is now a plan to create a methodically “humanized” mouse model for pharmacological and toxicological studies by replacing all mouse Cyp2, Cyp3, and Cyp4 genes with all human CYP2, CYP3, and CYP4 genes [214]. Future work in other models and non-models that similarly target enzyme-metabolism-associated gene families could be of high translational relevance for human health.

## Obtaining and maintaining universal gene family nomenclatures

The human gene mapping community began publishing proposals on standardized gene nomenclature in the 1970s “pre-genomics” era, e.g., [215, 216], and the HUGO Gene Nomenclature Committee (HGNC) has been naming human genes now for over 40 years [217]. However, these efforts had already been ongoing for mouse genetics since the 1940s [218]. Subsequent collaboration between the human and mouse gene nomenclature committee has enabled logical naming—and renaming—of many gene families to reflect evolutionary relationships. For example, the human CD300 gene family includes sequentially named genes that modulate a diverse array of immune responses, named CD300A–CD300H [219–221]. The original nomenclature for mouse models previously spanned a variety of root symbols including dendritic-cell-derived Ig-like receptor (DIgR), CRMF-35-like molecules (CLM), leukocyte mono-Ig-like receptors (LMIR), and myeloid-associated Ig-like receptors (MAIR) [219], but these mouse genes have since been renamed in line with the human genes using the Cd300 root. In more subtle cases, mismatches in nomenclature between closely related genes can be revealed through phylogenetic analyses; for instance, leukocyte receptor tyrosine kinase (LTK) in ray-finned fishes is a closer homolog of ALK receptor tyrosine kinase (ALK; previously anaplastic lymphoma kinase) in mammals than it is to LTK in mammals [74]. As sequencing efforts in models and non-models continue to accelerate, there is an urgent need to continue to standardize gene nomenclature not only within species, but also between species using phylogenetic criteria.

With regard to the standardization of nomenclature of gene families based on evolutionary divergence, the enzymic metabolism gene families offer one of the earliest examples, originating with the cytochrome P450 monooxygenase superfamily. From 1975 to 1985, leading scientists researching cytochrome P450 genes would convene at least once yearly, during the “Microsomes and Drug Oxidations” (MDO) or “P450 Biochemistry and Biophysics” symposia, to discuss what the best name might be for their (personal favorite) enzyme isolated in their respective laboratories. These enzymes (in human, rat, mouse, rabbit, pig, cow, chicken, fish, yeast, and *Pseudomonas putida*) are all membrane-bound (microsomal or mitochondrial) proteins, except for the bacterial cytosolic enzyme which is soluble.

With the advent of isolating mRNA from ribosomes treated with antibodies to these detergent-solubilized enzymes, followed by reverse transcriptase to isolate the cDNA, the deduced amino acid sequence of these membrane-bound proteins could be determined. Once it was discovered that these P450 genes encoded a consensus sequence of eight amino acids in the enzyme active site—a consensus found in at least ten organisms as diverse as human and a bacterium [222]—the logical solution to standardized nomenclature was to base P450 gene names on evolutionary divergence.

At first, Roman numerals were included [222, 223], but this clumsy approach was quickly discarded. A root symbol “CYP” (standing for cytochrome P450) was then agreed upon, with Arabic numerals for gene families, capital letters for subfamilies, and Arabic numerals again for individual members; no subscripts or superscripts are allowed in standardized gene nomenclature, and hyphens are only used in specific exceptions and usually to separate two unrelated consecutive numerals. Two CYP genes encoding proteins (from the same species) that showed < 40% amino acid sequence similarity were relegated to different gene families. Two CYP genes having ~ 40 to ~ 65% amino acid sequence similarity were assigned to the same family, whereas two genes encoding proteins that displayed ≥ 65% amino acid sequence similarity were listed as members of the same subfamily [224]. Gene symbols are in all capital letters for human and most vertebrate genomes, i.e*.* *CYP1A2*, *CYP1B1*, *CYP17A1*, *CYP51A1*, etc. Mice deviate from this model due to historical contingency of an earlier nomenclature, capitalizing only the first letter; hence, the mouse orthologous genes are named *Cyp1a2*, *Cyp1b1*, *Cyp17a1*, *Cyp51a1*, etc.

In the process of naming the CYP gene families, they were originally somewhat arbitrarily divided into different classes of organisms (Table 4), based on an assumption that the number of P450 genes thought likely to exist in all animals on the planet would not exceed 50. However, this assumption has proven to be a striking underestimate. As of July 13, 2022, a total of 125,326 CYP genes in 8455 gene families among vertebrates, protozoa, plants, fungi, eubacteria, archaebacteria, and viruses have been named [D R Nelson, personal communication]—with an anticipation that over one million will be reached. In general, plant genomes have far more P450 genes than animal genomes, because plant P450-mediated pathways are critical for virtually all life processes: growth, differentiation, defense (phytoalexin formation), fruit production, flower color, and formation of the attractive and repulsive scents of flowers [225].

**Table 4:** Historical format for the assignment of CYP gene families (https://drnelson.uthsc.edu/)

Gene family numbering	Class of organisms
CYP1–CYP49	Animals
CYP51–69	Yeast, Fungi
CYP71–99	Plants
CYP101–299	Bacteria, Viruses
CYP301–499	Animals
CYP501–699	Lower eukaryotes
CYP701–999	Plants
CYP1001–2999	Bacteria, Viruses
CYP3001–4999	Animals
CYP5001–6999	Lower eukaryotes
CYP7001–9999	Plants
CYP10001–29,999	Bacteria, Viruses*
CYP30001–49,999	(Animals)*
CYP50001–69,999	Lower eukaryotes

*No genes yet included in these ranges

Gene family numbers for other P450 genes were assigned in chronological sequence as they were identified (hence, CYP6, 9, 12 are insect gene families; *CYP10A1* is a pond snail gene; and *cyp-13A1* is a nematode gene, etc.). As a consequence, human CYP gene families are not named with sequential numbers, but rather CYP1, 2, 3, 4, 5, 7, 8, 11, 17, 19, 20, 21, 24, 25, 26, 27, 39, and 46 [213]. Although these authoritative efforts have yielded a stable nomenclature for this gene family, they also reflect a problem of sustainability. The herculean task of continually updating and maintaining a stable nomenclature of CYP genes—and specifically P450 genes—has been undertaken by a single curator, David R Nelson. As new genomes are published, the extraction and naming of all present P450 genes using BLAST searches (by DR Nelson) remains the predominant source of nomenclature updates for non-models. Dependency of nomenclature on single individuals augurs gene identification crises like the taxonomic identification crises lamented by current-day systematics [226]. Sustainable solutions to gene nomenclature are achievable through sustained funding and collaboration between gene nomenclature committees across multiple organisms and represent an exciting challenge and research opportunity that can integrate the expertise of gene taxonomists and computational biologists, aspirationally generating a well-defined and extensible nomenclature for comparative genomics.

As the CYP gene superfamily was developed during the late 1980s and early 1990s, dozens of other enzymatic metabolism gene families (e.g., Table 3) also began to establish standardized nomenclature systems based on evolutionary divergence. Work on these and many other gene families such as the olfactory receptors [227] by the HGNC and more recently also by the Vertebrate Gene Nomenclature Committee (VGNC, vertebrate.genenames.org), a sister project of the HGNC [228], has enabled the implementation of a systematic approach to standardized nomenclature based on naming paralogous genes originating from a common ancestor. However, the proliferation of genomes from non-model species now provides an opportunity to stabilize a consistent gene nomenclature at deeper taxonomic scales, as has already been instigated for cytochrome P450 genes, through collaboration between established nomenclature committees [229]. Other efforts have ensured that the immunoglobulin (*I*g) genes that encode antibodies across ray-finned fishes were recently standardized, because *I*g*T* and *I*g*Z* were found to be evolutionary forms of the same antibody that was independently discovered and named in different species [230]. This standardization across half of all living vertebrates demonstrates the utility of phylogenetic comparative genomics to gene nomenclature at such large taxonomic scales. As genome sequences continue to accrue, gene taxonomists are now poised to work with nomenclature committees to harness the power of this comparative framework and decode the history of paralog diversification in complex gene families, thereby stabilizing nomenclature across the Tree of Life.

## Veils of deep ancestry that limit our perception of gene family evolution

In general, investigations of gene families are rooted in comparative approaches. As genes of interest become identified, approaches based on sequence similarity or homology are used to detect similar genes within a target genome (intragenomically or intergenomically). Some of the most commonly used sequence similarity tools include BLAST [231, 232], PSI-BLAST [233, 234], diamond [235], and HMMER3 [236, 237] that collectively represent routine aspects of bioinformatic pipelines. Heuristic searches such as BLAST perform global and local alignments using dynamic programming algorithms (e.g., Needleman–Wunsch and Smith–Waterman algorithms) that require a database such as those hosted by NCBI, EMBL, or Ensembl for searching. The results are obtained based on a predetermined *e* value threshold, and the most similar matches are selected. However, *e* value-based metrics are not infallible, because they can give different results based on the completeness of the reference database. Similarity methods that apply *e* values are also vulnerable to inflating relatedness, as it is possible that only a small fraction of the query sequences is being considered in the analysis, rather than the whole sequence. This problem arises frequently in the annotation of non-model species when using highly curated databases as a reference (e.g., UniProtKB [238]). To complement *e* value-based identification, the identity of gene family members is often validated through phylogenetic analyses such as those conducted in IQTREE [239] or RevBayes [240].

Phylogenetic analyses allow candidate genes to be placed among known reference sequences to verify identification, assess the evolutionary history of gene gains and losses, and delimit unique gene expansions within lineages [59, 74]. However, it is well known that some genes are more amenable to phylogenetic inference than others [241–245], and the utility of each locus for evolutionary inference is dependent on the phylogenetic hypotheses being addressed [246–248]. Much of the heterogeneous utility of loci for phylogenetics can be attributed to variance in the rate of molecular evolution between sites, wherein fast-evolving sites can erode phylogenetic information [249, 250]. Just as phylogenetic information can be eroded by evolution, so can identification of homology [251]. In the presence of high rates of sequence evolution, “phylogenetic noise” in the data can mask or even mislead interpretation of the relationship of the queried gene family with the newly detected candidate genes in the database [251]. This problem arises as a natural consequence of numerous substitutions occurring in a gene over time and has the potential to promote erroneous inference [249, 252–254]. In cases with little true signal of evolutionary history, this phylogenetic noise can accumulate, leading to the estimation of phylogenetic tree topologies that appear to have strong statistical support [255–257]. These problems are not new to phylogenetics, and solutions and approaches to their mitigation should be adopted for studies of gene family evolution.

**Fig. 6:**
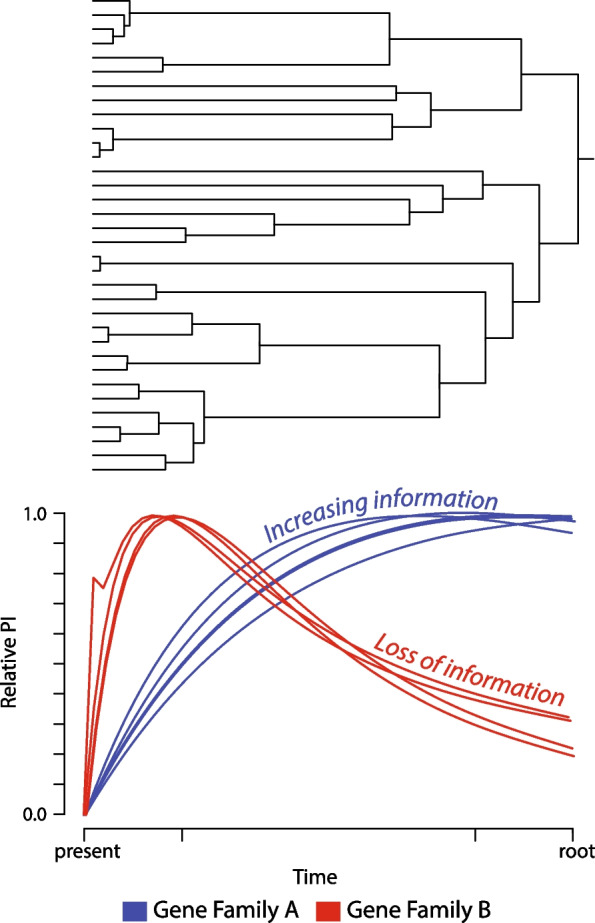
Concepts of the phylogenetic informativeness of gene families and the limits of ortholog detection. As lineages diversify (*top*), the rate of evolution of each lineage impacts the phylogenetic informativeness (PI) of each gene (*bottom*). In the case of gene families that exhibit relatively slower rates of sequence evolution, phylogenetic information content may continue to accrue over time, thereby increasing the amount of information available for inquiry (*blue*). In contrast, rapidly evolving loci can exhibit serial substitutions at the same site that erode phylogenetic information (*red*). The ability to resolve the evolutionary history of such “saturated” loci can be limited

The point at which noise (molecular changes reflecting chance convergence or parallelism) erodes signal (differences in nucleotides or amino acids since common ancestry) is not commonly accounted for—as an issue of statistical power—when defining the origins of genes. High rates of evolution at sites—especially genome-wide—will increase genetic distances between loci at deeper timescales [250, 258]. Consequently, an inability to determine which sites are evolving at rates predicted to contribute signal versus noise poses a major impediment in the selection of orthologs (Fig. 6). Common practices, such as using *e* value thresholds, do not address this problem [251]. Rates of evolution can vary between genes and lineages, and therefore cutoffs for accurate identification of orthologs differ between organismal groups and time scales [259].

Once genes become highly divergent, it becomes challenging—and then impossible—to assign homology of molecular sequence characters. However, quantifications of phylogenetic information loss are rarely incorporated into studies of homology. Quantification of evolutionary information loss through time requires consideration of the information gained by adding genetically distant taxa [250, 260, 261], characteristics of the sequence data (e.g., biases in nucleotide compositions [262, 263], amino acid versus nucleotide data [264], etc.), as well as molecular rate heterogeneity between taxa or loci [250, 255, 265, 266]. Such an integration facilitates calculation of the accumulation of signal versus loss of information due to chance convergences or parallelisms that provide an expectation of where power is highest for phylogenetic inference [246, 250, 267]. What is now needed is a mathematical framework that builds on such theory from phylogenetics, to predict when the inability to detect an ortholog would stem from a lack of phylogenetic information versus a likely true absence of an ortholog. This kind of framework will illuminate the predicted limits of ortholog detection that are critical to establishing where we are, and are not, confident in identifying the origins of genes in the human genome.

## The future of human genomics is comparative

Central to the diversification of the human genome are duplications of genes or entire genomic regions. Over short evolutionary timescales, gene birth events (i.e*.*, gene duplications that create paralogs) may provide redundancy for essential genes or increased levels of a gene product [268]. Over longer stretches of evolutionary time, these gene birth events provide the molecular basis for sequence evolution through neofunctionalization (i.e*.,* the acquisition of new functions), subfunctionalization (all functions of the original gene are maintained, but divided between the gene copies), or non-functionalization (accumulation of deleterious mutations resulting in gene death) [269, 270]. The function of specific gene duplications and clusters has been well studied in select species [55, 271]. However, the potential role of rapid paralog evolution in gene families as the substrate for further genomic novelty is only beginning to be explored. Testing for shifts in selection that have promoted the generation of new phenotypes in response to ecological opportunity is a fundamental aspect of macroevolution [272–274]. There is little doubt that the rapid expansion of a gene family can provide the necessary genomic foundation for pulses of phenotypic evolution. What remains unclear are the relationships between ecological opportunity, changes in selection, gene birth–death dynamics, and the timing of phenotypic diversification in the history of a lineage. Recent work has highlighted that large-scale genomic changes are temporally decoupled from the onset of phenotypic diversification within both the iconic adaptive radiations of African rift lake cichlids and Antarctic notothenioids [275, 276]. These findings highlight the critical, but often neglected, role of historical contingency in understanding adaptive evolution [277]. Therefore, as the taxonomic diversity of available genomes continues to increase, comparative studies with a wider taxonomic scope will be critical to determine the relationship between the tempo of gene family evolution and present-day phenotypes.

Successive tandem gene duplication events are of particular interest in clustered gene families. Relative to surrounding regions, clustered gene families often display disproportionately high levels of sequence and gene copy diversity. As such, clustered gene families may be hotspots for additional gene birth events, higher rates of gene death (loss), nucleotide substitution, sharing of regulatory sequences among multiple genes in tandem, exon swapping, and interlocus gene conversion. However, the diversification dynamics of gene clusters across vertebrate macroevolutionary history remains largely unexplored. What is needed is the continued development of theory [278] and novel tools [279, 280] that can be used to generate a unified paradigm for understanding the dynamics of clustered gene diversification both within and between species. As diversity within gene clusters contributes to a range of genetic disorders, such a paradigm is central to linking hyper-diverse clusters between model organisms and humans and might drive both diagnostic advances and the development of novel therapeutics.
